# Prediction of violent crime on discharge from secure psychiatric hospitals: A clinical prediction rule (FoVOx)

**DOI:** 10.1016/j.eurpsy.2017.07.011

**Published:** 2018-01

**Authors:** A. Wolf, T.R. Fanshawe, A. Sariaslan, R. Cornish, H. Larsson, S. Fazel

**Affiliations:** a Department of Psychiatry, University of Oxford, Warneford Hospital, Warneford Lane, OX3 7JX Oxford, UK; b Nuffield Department of Primary Care Health Sciences, University of Oxford, OX2 6GG Oxford, UK; c Department of Medical Epidemiology and Biostatistics, Karolinska Institutet, 171 77 Stockholm, Sweden; d Oxford Health NHS Foundation Trust, OX3 7JX Oxford, UK; e School of Medical Sciences, Örebro University, 701 82 Örebro, Sweden

**Keywords:** Forensic psychiatry, Violence, Psychometry and assessments in psychiatry, Risk assessment, Crime, Secure hospital, Clinical prediction

## Abstract

**Background:**

Current approaches to assess violence risk in secure hospitals are resource intensive, limited by accuracy and authorship bias and may have reached a performance ceiling. This study seeks to develop scalable predictive models for violent offending following discharge from secure psychiatric hospitals.

**Methods:**

We identified all patients discharged from secure hospitals in Sweden between January 1, 1992 and December 31, 2013. Using multiple Cox regression, pre-specified criminal, sociodemographic, and clinical risk factors were included in a model that was tested for discrimination and calibration in the prediction of violent crime at 12 and 24 months post-discharge. Risk cut-offs were pre-specified at 5% (low vs. medium) and 20% (medium vs. high).

**Results:**

We identified 2248 patients with 2933 discharges into community settings. We developed a 12-item model with good measures of calibration and discrimination (area under the curve = 0.77 at 12 and 24 months). At 24 months post-discharge, using the 5% cut-off, sensitivity was 96% and specificity was 21%. Positive and negative predictive values were 19% and 97%, respectively. Using the 20% cut-off, sensitivity was 55%, specificity 83% and the positive and negative predictive values were 37% and 91%, respectively. The model was used to develop a free online tool (FoVOx).

**Interpretation:**

We have developed a prediction score in a Swedish cohort of patients discharged from secure hospitals that can assist in clinical decision-making. Scalable predictive models for violence risk are possible in specific patient groups and can free up clinical time for treatment and management. Further evaluation in other countries is needed.

**Funding:**

Wellcome Trust (202836/Z/16/Z) and the Swedish Research Council. The funding sources had no involvement in writing of the manuscript or decision to submit or in data collection, analysis or interpretation or any aspect pertinent to the study.

## 1 Background

While psychiatric inpatient numbers have continued to be reduced in Western countries in the last two decades [1], [2], forensic psychiatry has seen the opposite trend and a recent overview found forensic psychiatric inpatient beds have increased steadily from 1990 to 2012 [3]. There are now over 7000 beds in England and Wales [4] and about a fifth of the mental health budget in England and Wales is spent on forensic psychiatric services [5]. Annual costs per patient are estimated at between €190,000 in low secure and €340,000 in high secure hospitals [4].

One of the key justifications for such high costs has been that forensic psychiatric patients are at increased risk of repeat violence on release from hospital compared to general psychiatric patients and therefore their treatment should address a wide range of needs. A recent systematic review found studies from three European countries, showing high rates of violent offending following discharge from secure hospitals in England & Wales (7 studies; 1589 to 8403 per 100,000 person–years) [6], Sweden (3 studies; 1041 to 3019 per 100,000 person–years), and Norway (one study; 486 per 100,000 person–years). Absolute risks of reconviction for grave offences (that could potentially attract life sentences) following discharge are around 7% within two years of discharge, as found in two recent representative studies from the UK [7], [8].

Current approaches to reduce violence risk generally involve structured risk assessment tools allied to clinical decision-making, with over 90% of medium secure forensic units in England using one or more such tools [9] and their use is recorded as a key service outcome [10]. Such approaches are resource intensive and time consuming, taking around 16 person–hours for the first assessment [11] and many hours for subsequent ones, with limited accuracy [12], authorship bias in their reporting [13] and considerable variation in what constitutes ‘high risk’ [14], so that using such categorisations in current tools has questionable usefulness [15]. Furthermore, they are typically developed in non-psychiatric samples and their external validity is worse in forensic psychiatric populations [16]. Scalable tools in general psychiatry have been developed although not widely adopted [17], [18].

Therefore, we have developed a simple, free, scalable tool to assess the risk of violence in patients discharged from secure and forensic psychiatric hospitals, using routinely collected data.

## 2 Methods

### 2.1 Study sample

We conducted a longitudinal cohort study of all individuals aged 15–65 discharged from secure and forensic psychiatric hospitals into the community between 1992 and 2013 through linkage of population-based registers in Sweden. The final study cohort consisted of all discharged individuals, with a single discharge for each patient, selected at random, with equal probability. Repeat discharges complicate model fitting and interpretation and were excluded. Each individual was followed from the day of discharge until first violent offending, death, emigration or end of follow-up (12 or 24 months post-discharge). If an individual was rehospitalised without a reoffence, this did not end follow-up as we included crimes committed during rehospitalisation. The study was approved by the Regional Ethics Committee at Karolinska Institutet.

### 2.2 Measurement of risk factors

Data from several national registers were linked to obtain information on risk factors, with unique personal identification numbers enabling accurate linkage [19]. Sociodemographic factors were obtained from the Total Population Register [20] and the Longitudinal Integration Database for Health Insurance and Social Studies. From the National Crime Register, we obtained information on any previous violent crime conviction. In line with previous work, violent crime was defined as homicide, assault, robbery, arson, any sexual offence, or threats and harassment [21]. Serious violent crime was defined as homicide, aggravated assault, aggravated robbery, rape, sexual coercion or sexual exploitation. We identified diagnoses of psychiatric disorders and substance use disorders from the National Patient Register (see Appendix for all risk factor definitions).

### 2.3 Measurement of outcomes

Our primary outcome was the occurrence of violent offending within 24 months of discharge from hospital, with 12 months post-discharge a secondary outcome. Repeat offences by an individual within these two years were not considered. Conviction data were used because the Swedish criminal code determines that individuals are convicted as guilty regardless of mental disorder, although sentencing may be informed by mental disorder and no plea-bargaining is permitted at the conviction stage. Violent crime was defined as above.

### 2.4 Statistical methods

Statistical analysis was based on Cox regression, adjusting for risk factors as described below.

#### 2.4.1 Adjustment for risk factors

Based on existing evidence into criminal history, sociodemographic and clinical factors [22], [23], we grouped variables a priori on the anticipated strength of association with the outcome in decreasing levels of priority [24], [25]. All variables were categorised in this way in a protocol before any statistical analysis was carried out (see below for description of variable groups). Table 1 specifies the group to which each variable was assigned.

**Table 1:** Baseline characteristics and variable grouping for a cohort of secure psychiatric patients.

Variable	*n* = 2248	Group
*Sex (male)*	1938 (86%)	1
*Age at discharge (IQR)*	36 (29–45)	1
*Previous violent crime*	1836 (81.7%)	1
*Previous serious violent crime*	590 (26.2%)	1
*Primary diagnosis at discharge*		1
Schizophrenia-spectrum	944 (45.7%)	
Bipolar disorder	130 (6.3%)	
Unipolar depression	97 (4.7%)	
Anxiety disorders	139 (6.7%)	
Other^a^	754 (36.5%)	
*Drug use disorder at hospitalisation or discharge*	540 (26.2%)	1
*Alcohol use disorder at hospitalisation or discharge*	219 (10.6%)	1
*Personality disorder at discharge*	563 (27.3%)	1
*Educational level*		2
Lower secondary	1084 (54.1%)	
Upper secondary	819 (40.8%)	
Post secondary	102 (5.1%)	
*Marital status (single)*	1648 (74.4%)	2
*Employment before admission*	171 (7.6%)	2
*Number of previous inpatient episodes (five or more)*	1110 (52.6%)	2
*Previous forensic inpatient episode (one or more)*	755 (33.6%)	2
*Lifetime drug use disorder*	1050 (49.0%)	2
*Lifetime alcohol use disorder*	780 (34.7%)	2
*Length of stay in forensic hospital (12 months or more)*	986 (43.9%)	2

Primary diagnosis, drug use and alcohol use disorders at hospitalisation or discharge, and personality disorder had 8.2% of missing data. Educational level had 10.8% missing, marital status 1.4%, number of previous inpatient episodes 6.2%, lifetime drug use disorder 4.6%, and lifetime alcohol use disorder 5.7%.

a In the ‘other’ group, 356 (47.2%) had a primary diagnosis of personality disorder, 152 (20.2%) alcohol or drug use disorder, 49 (6.5%) autism spectrum disorder.

#### 2.4.2 Risk factor groups

Group 1 consists of variables thought necessary to include in the statistical model regardless of statistical significance, in order to ensure face validity and to reduce the number of candidate predictors used in the variable selection procedure described below. For the majority of these risk factors, there was evidence from previous research of an association with the outcome measure. We drew on systematic reviews of risk factors for violence in patients with severe mental illness for this information [22].

Group 2 consists of variables thought likely to show an association with outcomes, but which are not required to be included to achieve face validity. These variables were included in a backwards stepwise selection procedure, with group 1 variables always retained in the model, such that they were sequentially rejected in order of *P*-value until no group 2 variables remained with *P*-values greater than 0.1.

Continuous variables were included in the model as linear terms as there was not strong evidence of departure from linearity between continuous variables and the log-odds of the outcome. Interactions between risk factors were not considered.

#### 2.4.3 Missing data

Missing data was imputed via multiple imputation using chained equations (with twenty imputations) using a regression model that used as explanatory variables all other risk factors that were candidates for inclusion in the model, and the outcome variable [26]. Estimates of coefficients in the final prediction rule were obtained by pooling across imputations, using standard methodology [27].

#### 2.4.4 Internal validation and goodness of fit

The internal validity of the model was assessed using bootstrapping to assess its predictive accuracy [28]. Bootstrapping was used to create 100 samples drawn with replacement from the data set. Predictive accuracy was summarised using the following measures:

• the concordance index [29] to assess discrimination (ability of the model to distinguish between those who do and do not commit a violent crime, with a value of one meaning perfect discrimination);
• the Brier score [30] for calibration (model goodness of fit–whether the predicted risk is systematically off target, with zero meaning perfect calibration); the Brier score measures the mean squared difference between the predicted probability and the actual outcome (violent crime or no violent crime);
• sensitivity, specificity, positive predictive value, and negative predictive value based on the 5% and 20% thresholds of predicted probability at 12 and 24 months post-discharge [6].

These measures were calculated using the predicted probabilities obtained by averaging the predictions from each of the multiply imputed datasets, each applied to the final model. Pre-specified cut-offs were informed by a systematic review of 15 studies on violent offending following discharge from forensic psychiatric hospitals [6], that reported a pooled rate of 3900 per 100,000 person–years or around 4% per year. The proportional hazards assumption was tested using stratified Kaplan–Meier survival curves and the Grambsch and Therneau test [31]. The proportions of predicted and observed events at different levels of predicted probability were compared using a calibration plot.

#### 2.4.5 Sensitivity analyses

We performed two sensitivity analyses, which were not pre-specified in the protocol. First, we refitted the final model using only discharges in 2001 or later (introduction of ICD-10) to examine differences in the effects of risk factors due to secular trends or reporting differences. Second, we refitted the model in those under 40 only, as some sociodemographic variables may be have been recorded differently in older patients. Additionally, we conducted exploratory risk factor interaction analyses, using a Bonferroni-corrected level of significance of *P* = 0.0005.

### 2.5 Web calculator

We applied the model coefficients to develop a web calculator called Forensic Psychiatry and Violence tool Oxford (FoVOx), which is free to use. This provides both a risk classification (low [< 5%], medium [5–20%], high [≥ 20%]; based on 24 month violent offending risk) and a probability of violent offending within the next 12 or 24 months.

Stata (version 12) and R version 3.2.1 were used for all analyses. The TRIPOD statement was followed (Appendix) [32].

## 3 Results

We identified a cohort of 2248 forensic psychiatric patients with 2933 discharges into community settings between 1 January 1992 and 31 December 2013, with 155 (6.9%) patients with violent offences within 12 months, and 244 (10.9%) within 24 months; 34 (1.5%) committed a serious violent crime within 24 months (Appendix Table 1 for types of crime pre- and post-discharge). The median age at discharge was 36 years and 86% of the cohort were male (Table 1 for baseline characteristics).

Risk factors included in the final model were age at discharge, male sex, previous violent crime, previous serious violent crime, primary diagnosis at discharge, drug use disorder at hospitalisation or discharge, alcohol use disorder at hospitalisation or discharge, personality disorder diagnosis at discharge, employment before admission, five or more previous inpatient episodes, lifetime drug use disorder, and one or more years length of stay. The strongest predictors were previous violent crime (hazard ratio [*HR*]: 3.2; 95% confidence interval [CI] 2.3 to 4.5) and sex (female vs. male HR: 0.4, 95% CI 0.3 to 0.6 (Table 2). Previous serious violent crime was associated with a lower risk than non-serious violent crime, but a doubling compared to no violent crime (as serious violent crime is a subset of all violent crime). The model showed good overall discrimination over the total follow-up (concordance index: 0.73). We found no significant differences in risk factors after conducting sensitivity analyses including only discharges post-2001 (Appendix Table 2), or those under 40 (Appendix Table 3).

**Table 2:** Associations between risk factors and violent crime in the derivation sample from the multiple regression model (after multiple imputation).

Variable	*Hazard ratio* (95% CI)	*P*-value
*Sex (female)*	0.43 (0.29–0.64)	< 0.001
*Age at discharge*	0.97 (0.96–0.98)	< 0.001
*Previous violent crime*	3.22 (2.28–4.53)	< 0.001
*Previous serious violent crime*	0.64 (0.51–0.80)	< 0.001
*Primary diagnosis at discharge*		
Schizophrenia spectrum	1.00 (ref)	n/a
Bipolar disorder	1.82 (1.24–2.66)	0.002
Unipolar depression	1.33 (0.83–2.14)	0.234
Anxiety disorders	1.12 (0.72–1.74)	0.610
Other	1.36 (1.06–1.73)	0.014
*Drug use disorder at hospitalisation or discharge*	0.89 (0.69–1.15)	0.366
*Alcohol use disorder at hospitalisation or discharge*	1.26 (0.94–1.67)	0.116
*Personality disorder at discharge*	1.36 (1.09–1.69)	0.006
*Employment before admission*	0.56 (0.37–0.86)	0.007
*Number of previous inpatient episodes (five or more)*	0.63 (0.51–0.77)	< 0.001
*Lifetime drug use disorder*	2.22 (1.71–2.87)	< 0.001
*Length of stay in forensic hospital (12 months or more)*	0.63 (0.52–0.77)	< 0.001

**Fig. 1:**
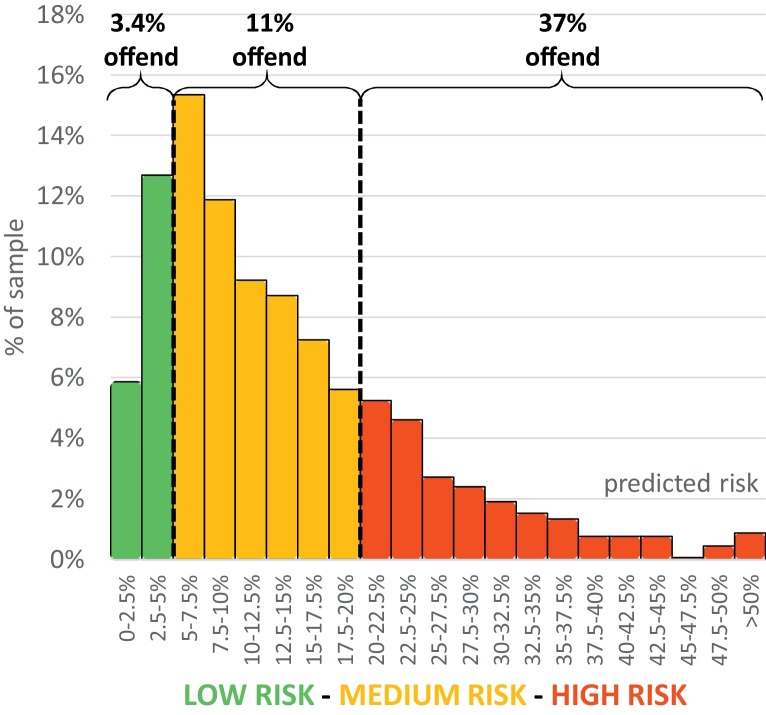
Observed and predicted risk of violent crime at 24 months, by risk categorisation.

For the risk of violent offending at 24 months after discharge, using the 5% cut-off (low to medium), sensitivity was 96% and specificity was 21%. Positive and negative predictive values were 19% and 97%, respectively. Using the 20% cut-off (medium to high), sensitivity was 55%, specificity 83% and the positive and negative predictive values were 37% and 91%, respectively. The concordance index (AUC) was 0.77 (Appendix Fig. 1) and the Brier score (*Br*: 0.0876) was lower than that using the mean predicted probability (*Br*: 0.0985) or using a predicted probability of zero, i.e. classifying all individuals as low risk (*Br*: 0.1108). In the low, medium and high risk groups, 3%, 11% and 37% had a violent offence within 24 months (Fig. 1).

For the risk of violent offending at 12 months after discharge, using the 5% cut-off, sensitivity was 88% and specificity was 44%. Positive and negative predictive values were 13% and 97%, respectively. Using the 20% cut-off, sensitivity was 22%, specificity 96% and the positive and negative predictive values were 34% and 93%, respectively. The concordance index (AUC) was 0.77 (Appendix Fig. 1), and the Brier score (*Br*: 0.0607) was lower than that using the mean predicted probability (*Br*: 0.0657) or using zero (*Br*: 0.0707).

Calibration plots indicate adequate calibration of the predicted probabilities against observed proportions of violent offending at 12 and 24 months (Appendix Fig. 2). Bootstrapping showed good predictive accuracy at both 12 and 24 months (Table 3), though sensitivity dropped slightly. Two by two tables comparing predicted and observed outcomes are presented in Appendix Table 4. Out of 97 possible interaction effects, one was significant at the Bonferroni-corrected significance level of *P* = 0.0005 (Appendix Table 5).

**Table 3:** Internal validation, comparing model performance with 100 samples drawn with replacement (bootstrapping).

		12 months		24 months	
Cut-off	Measure	Model (%)	Bootstrapped Mean (95% CI)	Model (%)	Bootstrapped Mean (95% CI)
5%	Sensitivity	88	86% (85–87)	96	95% (94–95)
	Specificity	44	46% (44–47)	21	24% (23–25)
20%	Sensitivity	22	22% (20–24)	55	51% (49–53)
	Specificity	96	95% (94–95)	83	82% (81–83)

### 3.1 Web calculator

A beta version of the online risk calculator for violent offending (based on the coefficients in Appendix Table 6) can be found at http://oxrisk.com/fovox. If missing values were present, this calculator reports the upper and lower range of estimates of risk allowing for these missing variables.

## 4 Discussion

We have developed a prediction model for the risk of violent offending after discharge from secure (or forensic) psychiatric hospitals. The model demonstrated good measures of discrimination and calibration, and was used to develop an online tool (FoVOx) that is free, scalable, and easy to use.

### 4.1 Clinical implications

Our model identifies around a fifth of patients as low risk (defined as individuals with < 5% of violent crime within two years of discharge), of which only 3% offended within 24 months of discharge. The ‘prevention paradox’ (where a majority of adverse outcomes occur in those considered low risk, in part because most people find themselves in that category) has also been cited as a criticism against violence risk assessment [15]. However, at 24 months post-discharge, our model correctly identified 55% of offenders as high risk and, of all those classified as high risk, 37% did subsequently offend. Furthermore, the use of the actuarial score allows for good discrimination between individual patients and could be used for treatment matching. At both 12 months and 24 months, we reported a concordance index of 0.77. This means that in 77% of discordant pairs (where one offends and the other one does not), FoVOx would assign a higher risk to the former.

Using a simple tool can potentially free up clinical time to treat and manage violence risk in this patient group [33]. Promising interventions to reduce the risk of violence include treatment of comorbidities and other modifiable risk factors. For example, treating substance use disorders through therapeutic community interventions after discharge may reduce reoffending [34].

In our model, previous serious violent crime was associated with a smaller increase in risk (doubling) than any violent crime (tripling) compared to no violent crime, which is consistent with some earlier work that finds that very serious offences, such as homicide, are not correlates of recidivism [35]. A length of stay of 12 months or more was found to be protective (adjusting for all other factors in the model, including age) and likely to be subject to post-discharge statutory supervision. Our finding that five or more previous inpatient episodes was associated with a lower risk of violence suggests that these patients are known to services and therefore interventions can be put in place before severe relapses.

### 4.2 Strengths

Risk assessment will need to be linked to management to improve patient outcomes and future work will need to examine how this can be most effectively done. However, compared to current risk assessment approaches, FoVOx has some advantages. First, it uses robust methodology, including its sample size of over 2000 individuals, the total cohort of those discharged from secure hospitals in Sweden between 1992 and 2013. We used a design, cut-offs, risk factors, and internal validation that were pre-specified in a protocol before any analyses were performed. Second, it has been developed specifically in forensic psychiatric patients, whereas other common approaches have been developed using heterogeneous samples from criminal justice and forensic psychiatry, and risk factors and baseline risks differ from prison [36] or general psychiatry populations [2]. Hence, it is not surprising that field studies show considerable shrinkage in the predictive accuracy of tools such as the HCR-20 in forensic samples [37]. Third, there may be clinical benefits of a freely available and quicker risk assessment in that resources can be redirected towards clinical care and risk management. More resource intensive forms of risk assessment could be limited to those scoring higher in FoVOx. Further, psychiatric services in countries without the resources required for training and other costs of current approaches will likely benefit from a risk score to support clinical judgement. Finally, all included risk factors were from routinely collected register data and are likely to be known for most patients without additional interviewing; some items can be marked as unknown in the FoVOx calculator if they are unavailable. If one or more items are marked as unknown, FoVOx provides a risk range, based on the lower and upper bound of possible answers.

The performance of FoVOx is typically better than other tools used in forensic psychiatry, which show AUCs for any violence within 12 months of discharge of 0.70 or less, compared to 0.77 for FoVOx [38]. Similarly, FoVOx performs no worse when compared to a wide-ranging review of such instruments used in criminal justice and forensic psychiatry (median AUC: 0.72) [12], including the Medium Security Recidivism Assessment Guide [39].

### 4.3 Limitations

One limitation is the use of mostly static risk factors, and FoVOx should not be used to monitor within-individual changes in risk, for which other tools may be more appropriate [40], [41]. Another limitation is that, due to the small number of individuals in secure psychiatric hospitals in Sweden, it was not possible to perform an external validation of the model. Though bootstrapping showed good predictive accuracy in internal validation, FoVOx will need to be validated in different samples, in particular as other jurisdictions will have different legal frameworks with which to detain mentally disordered offenders. However, Sweden and England have similar provisions for individuals at higher risk. In Sweden, about two thirds of forensic psychiatric patients are under ‘special court supervision’ which means that they cannot be discharged without court approval [42]. In England and Wales, restriction orders (under Sections 41 or 49 of the Mental Health Act) can be used to supplement hospital detention and, in 2015, there were around 4600 of these (which amounts to around 60% of the total forensic psychiatric population) [43]. Additionally, due to the low number of post-discharge serious violent crimes, it was not possible to assess the performance of our model in predicting serious violence. Another issue is the effect of risk factors and univariate analyses from two large UK-based studies find similar associations, including for age, sex, length of stay, substance use disorders and psychiatric diagnoses [8], [44].

The ‘ceiling effect’, the idea that we have reached a plateau in the performance of risk assessment, suggests that optimising such tools has limited potential. Future research into psychological, genetic or epigenetic risk factors, or dynamic monitoring, may raise this ceiling. However, until such a time, the emphasis should be on reaching the ceiling in the most cost-effective way. Tools like FoVOx show similar or better performance to other tools, but are easier, quicker, and free to use whilst at the same time being scalable, fully transparent, and less subjective. Additionally, while measures of interrater reliability of structured clinical judgement tools are generally high in research settings [45], this may not be the case when used in adversarial settings [46].

How FoVOx can be incorporated into clinical practice will require feasibility and acceptability studies, in discussion with clinicians. It is possible that the probability scores provided can be used as evidence to external bodies that require this information, such as mental health tribunals, and also in the transition from forensic to general psychiatric services where evidence of low risk may need demonstrating in different ways, including risk scores. Assuming that these tools are unlikely to reach beyond AUCs of 0.80, research focus should move to risk management. Randomized controlled trial evidence of the effectiveness of risk assessment in reducing violence is currently limited to one study [47]. Therefore, research should move beyond optimising tools for risk assessment, and implement free and simple risk tools. New work should focus on risk management that is linked to interventions to reduce risks, such as treating comorbid substance use disorders [48], and improving treatment adherence [49].

## Funding

This work was supported by a grant from the Wellcome Trust (202836/Z/16/Z), and the Swedish Research Council.

## Disclosure of interest

HL has served as a speaker for Eli-Lilly and Shire and received research grants from Shire, all outside the submitted work. SF has received one speaker's fee from Janssen outside of the submitted work.

The authors A.W., T.R.F., A.S. and R.C. declare that they have no competing interest.
