# Supplementary material for: Prediction of violent crime on discharge from secure psychiatric hospitals: A clinical prediction rule (FoVOx)
Source: Eur Psychiatry. 2018 Jan;47:88–93. doi: 10.1016/j.eurpsy.2017.07.011 (PMC5797975; doi:10.1016/j.eurpsy.2017.07.011)
Supplement: Supplementary file 1 [file mmc1.doc]

**Appendix**

**Risk factor definitions**

From the National Crime Register, we obtained information on any previous violent crime conviction (binary) and any previous serious violent crime conviction (binary). In line with previous work, violent crime was defined as homicide, assault, robbery, arson, any sexual offence (rape, sexual coercion, child molestation, indecent exposure, or sexual harassment), or threats and harassment. Serious violent crime was defined as homicide, aggravated assault, aggravated robbery, aggravated arson, rape, sexual coercion, or sexual exploitation. From the Longitudinal Integration Database for Health Insurance and Social Studies, we obtained socio-economic factors at the year of patient episode. Civil status (ever married versus never married) was classified as binary variables. Number of years in education was recoded as a categorical variable.

We identified diagnoses of psychiatric disorders from the National Patient Register, which provides diagnoses for all inpatient psychiatric hospital admissions in Sweden since 1973 and outpatient care since 2001, according to the International Classification of Diseases (Eighth Revision [ICD-8], 1973-1986; Ninth Revision [ICD-9], 1987-1996; or Tenth Revision [ICD-10], 1997-2009).

We investigated the following specific/groups of psychiatric disorders: (1) primary diagnosis at discharge - as schizophrenia-spectrum disorders (ICD-9: 295, 297, 298 ; ICD-10: F20-F29), bipolar disorders (296 excl. 296D; F30-F31), unipolar depression (296B, 300E, 311; F32-F34.1), Anxiety disorder (300 excl. 300E, 309 ; ICD-10: F41, F43, F44, F45, F48), and other primary diagnoses (2) drug use disorder at hospitalization or discharge (292, 304, 305X; F11-F19), (3) alcohol use disorder at hospitalization or discharge (291, 303, 305A; F10), (4) diagnosis of personality disorder at discharge (301; F60-F62) (5) number of previous inpatient episodes (≥ 5 episodes, or < 5), (6) one or more previous forensic inpatient episodes, (7) any drug use disorder (lifetime), (8) any alcohol use disorder (lifetime), and (9) length of stay of current forensic hospital episode (≥1 year vs. <1 year).

**Appendix table** 1. Number of individuals with violent crime pre- and post-discharge

| **Type of offence** | **Pre-discharge** | **Post-discharge** |
| --- | --- | --- |
| **Any violent crime** | **1,836 (81.7%)** | **244 (10.9%)** |
| **Serious violent crime** | **590 (26.2%)** | **34 (1.5%)** |
| - Homicide and attempted homicide | 253 (11.3%) | 8 (0.4%) |
| - Aggravated assault | 207 (9.2%) | 20 (0.9%) |
| - Aggravated robbery | 24 (1.1%) | 0 (0%) |
| - Rape, sexual coercion, sexual exploitation | 130 (5.8%) | 6 (0.3%) |
| **Other violent crime** | **1,470 (65.4%)** | **211 (9.4%)** |
| - Common assault | 678 (30.2%) | 98 (4.4%) |
| - Assaulting an officer | 390 (17.3%) | 66 (2.9%) |
| - Other sexual offences | 79 (3.5%) | 7 (0.3%) |
| - Robbery | 125 (5.6%) | 14 (0.6%) |
| - Arson | 21 (0.9%) | 0 (0%) |
| - Threats and harassment | 771 (34.3%) | 111 (4.9%) |
| **None** | **412 (18.3%)** | **2,004 (89.1%)** |

Note : Some cases committed more than one type of crime.
*First crime within 2 years of discharge

**Appendix Table 2. Sensitivity analysis: Associations between risk factors and violent crime in the derivation sample from the multiple regression model (after multiple imputation), discharges from 2001 only**

| **Variable** | **Hazard ratio (95% CI)** | **p-value** |
| --- | --- | --- |
| Sex (female) | 0.38 (0.21-0.69) | 0.001 |
| Age at discharge | 0.97 (0.95-0.98) | <0.001 |
| Previous violent crime | 2.80 (1.60-4.89) | <0.001 |
| Previous serious violent crime | 0.69 (0.50-0.96) | 0.026 |
| Primary diagnosis at discharge   - Schizophrenia spectrum - Bipolar disorder - Unipolar depression - Anxiety disorders - Other | 1.00 (ref)  2.11 (1.14-3.89)  0.95 (0.34-2.67)  1.27 (0.69-2.33)  1.59 (1.15-2.18) | n/a  0.017  0.925  0.444  0.005 |
| Drug use disorder at hospitalisation or discharge | 1.07 (0.75-1.53) | 0.716 |
| Alcohol use disorder at hospitalisation or discharge | 1.32 (0.84-2.07) | 0.233 |
| Personality disorder at discharge | 1.43 (1.04-1.97) | 0.027 |
| Employment before admission | 0.63 (0.31-1.25) | 0.184 |
| Number of previous inpatient episodes (five or more) | 0.68 (0.50-0.91) | 0.009 |
| Lifetime drug use disorder | 2.45 (1.66-3.62) | <0.001 |
| Length of stay in forensic hospital (12 months or more) | 0.70 (0.52-0.93) | 0.013 |

**Appendix Table 3. Sensitivity analysis: Associations between risk factors and violent crime in the derivation sample from the multiple regression model (after multiple imputation), age <40 at discharge only**

| **Variable** | **Hazard ratio (95% CI)** | **p-value** |
| --- | --- | --- |
| Sex (female) | 0.39 (0.24-0.63) | <0.001 |
| Age at discharge | 0.98 (0.96-1.00) | 0.042 |
| Previous violent crime | 2.99 (2.01-4.44) | <0.001 |
| Previous serious violent crime | 0.55 (0.42-0.72) | <0.001 |
| Primary diagnosis at discharge   - Schizophrenia spectrum - Bipolar disorder - Unipolar depression - Anxiety disorders - Other | 1.00 (ref)  0.98 (0.54-1.79)  0.91 (0.48-1.74)  1.07 (0.65-1.76)  1.19 (0.90-1.58) | n/a  0.957  0.781  0.780  0.219 |
| Drug use disorder at hospitalisation or discharge | 0.89 (0.65-1.22) | 0.461 |
| Alcohol use disorder at hospitalisation or discharge | 1.17 (0.81-1.69) | 0.392 |
| Personality disorder at discharge | 1.55 (1.20-1.99) | 0.001 |
| Employment before admission | 0.48 (0.27-0.84) | 0.011 |
| Number of previous inpatient episodes (five or more) | 0.61 (0.48-0.78) | <0.001 |
| Lifetime drug use disorder | 2.10 (1.52-2.89) | <0.001 |
| Length of stay in forensic hospital (12 months or more) | 0.67 (0.54-0.85) | 0.001 |

**Appendix Figure 1. Model discrimination shown by receiver operating characteristics (ROC) curves**

**Appendix Figure 2. Calibration plots comparing predicted and observed risks of violent crime**

**12 months 24 months**

**Appendix Table 4. Two by two tables used to derive estimate of sensitivity, specificity, and positive and negative predictive values (after multiple imputation)**

|  |  | **12 months^1^** | | | **24 months^2^** | | |
| --- | --- | --- | --- | --- | --- | --- | --- |
| **Cut-off** |  | **Outcome +** | **Outcome -** | **Total** | **Outcome +** | **Outcome -** | **Total** |
| **5%** | **Prediction +** | 140 | 931 | 1071 | 239 | 1050 | 1289 |
|  | **Prediction -** | 19 | 727 | 746 | 10 | 284 | 294 |
|  | **Total** | 159 | 1658 | 1817 | 249 | 1334 | 1583 |
| **20%** | **Prediction +** | 35 | 69 | 104 | 138 | 232 | 370 |
|  | **Prediction -** | 124 | 1589 | 1713 | 111 | 1102 | 1213 |
|  | **Total** | 159 | 1658 | 1817 | 249 | 1334 | 1583 |

^1^Based on those with full year follow-up or event (n=1817); ^2^Based on those with full two year follow-up or event (n=1583)

**Appendix Table 5**. Risk factor interactions

|  | **Sex** | **Age at discharge** | **Previous violent crime** | **Previous serious violent crime** | **Primary diagnosis at discharge** | **DUD hospitalisation or discharge** | **AUD hospitalisation or discharge** | **Personality disorder at discharge** | **Employment before admission** | **5+ previous inpatient episodes** | **Lifetime DUD** |
| --- | --- | --- | --- | --- | --- | --- | --- | --- | --- | --- | --- |
| **Age at discharge** | 1.01  (0.97-1.05) p = 0.666 |  |  |  |  |  |  |  |  |  |  |
|  |  |  |  |  |  |  |  |  |  |  |  |
|  |  |  |  |  |  |  |  |  |  |  |  |
|  |  |  |  |  |  |  |  |  |  |  |  |
| **Previous violent crime** | 1.42  (0.54-3.76)  p = 0.475 | 1.00  (0.97-1.04)  p=0.816 |  |  |  |  |  |  |  |  |  |
|  |  |  |  |  |  |  |  |  |  |  |  |
|  |  |  |  |  |  |  |  |  |  |  |  |
|  |  |  |  |  |  |  |  |  |  |  |  |
| **Previous serious violent crime** | 1.35  (0.56-3.29)  p = 0.505 | 1.01  (0.98-1.03)  p = 0.589 | n/a |  |  |  |  |  |  |  |  |
|  |  |  |  |  |  |  |  |  |  |  |  |
|  |  |  |  |  |  |  |  |  |  |  |  |
|  |  |  |  |  |  |  |  |  |  |  |  |
| **Primary diagnosis at discharge** | 0.66 (0.13-3.32); 0.613 | 1.07 (1.03-1.11); 0.0003 | 1.10 (0.35-3.50); 0.866 | 0.50 (0.14-1.77); 0.284 | **Bipolar** |  |  |  |  |  |  |
|  | 0.23 (0.03-1.97); 0.181 | 1.03 (0.98-1.09); 0.265 | 2.06 (0.42-10.08); 0.372 | 1.63 (0.65-4.10); 0.301 | **Depression** |  |  |  |  |  |  |
|  | 1.56 (0.40-6.15); 0.526 | 1.04 (0.99-1.09); 0.093 | 0.47 (0.15-1.52); 0.207 | 0.83 (0.32-2.18); 0.706 | **Anxiety** |  |  |  |  |  |  |
|  | 0.33 (0.13-0.83); 0.018 | 1.02 (0.99-1.04); 0.127 | 1.29 (0.57-2.92); 0.538 | 0.99 (0.59-1.65); 0.969 | **Other** |  |  |  |  |  |  |
| **DUD at hospitalisation or discharge** | 0.40  (0.13-1.24)  p = 0.111 | 1.00  (0.98-1.02)  p = 0.948 | 1.26  (0.58-2.74)  p = 0.560 | 1.17  (0.73-1.87)  p = 0.518 | 2.21 (0.91-5.39); 0.080 |  |  |  |  |  |  |
|  |  |  |  |  | 1.20 (0.37-3.83); 0.760 |  |  |  |  |  |  |
|  |  |  |  |  | 1.91 (0.78-4.70); 0.159 |  |  |  |  |  |  |
|  |  |  |  |  | 1.30 (0.83-2.02); 0.248 |  |  |  |  |  |  |
| **AUD at hospitalisation or discharge** | 1.77  (0.69-4.53)  p = 0.236 | 1.00  (0.97-1.03)  p = 0.933 | 0.70  (0.28-1.80)  p = 0.465 | 1.64  (0.92-2.94)  p = 0.095 | 0.97 (0.30-3.16); 0.957 | 0.60  (0.34-1.06)  p = 0.077 |  |  |  |  |  |
|  |  |  |  |  | 1.03 (0.31-3.40); 0.964 |  |  |  |  |  |  |
|  |  |  |  |  | 0.40 (0.05-3.10); 0.381 |  |  |  |  |  |  |
|  |  |  |  |  | 1.12 (0.60-2.09); 0.715 |  |  |  |  |  |  |
| **Personality disorder at discharge** | 0.60  (0.27-1.34)  p = 0.212 | 0.98  (0.96-1.01)  p = 0.145 | 0.61  (0.29-1.24)  p = 0.172 | 0.76  (0.49-1.18)  p = 0.216 | 0.71 (0.27-1.86); 0.482 | 1.20  (0.80-1.78)  p = 0.381 | 0.88  (0.51-1.52)  p = 0.645 |  |  |  |  |
|  |  |  |  |  | 0.38 (0.14-1.07); 0.067 |  |  |  |  |  |  |
|  |  |  |  |  | 1.37 (0.56-3.36); 0.489 |  |  |  |  |  |  |
|  |  |  |  |  | 0.79 (0.49-1.28); 0.341 |  |  |  |  |  |  |
| **Employment before admission** | 1.80  (0.40-8.13)  p = 0.443 | 1.03  (0.99-1.08)  p = 0.180 | 0.66  (0.23-1.89)  p = 0.437 | 1.01  (0.39-2.63)  p = 0.983 | 1.61 (0.36-7.14); 0.530 | 1.25  (0.43-3.61)  p = 0.676 | 0.96  (0.33-2.81)  p = 0.935 | 0.63  (0.26-1.55)  p = 0.315 |  |  |  |
|  |  |  |  |  | 0.30 (0.03-3.03); 0.304 |  |  |  |  |  |  |
|  |  |  |  |  | 0.48 (0.05-4.98); 0.541 |  |  |  |  |  |  |
|  |  |  |  |  | 1.19 (0.32-4.44); 0.792 |  |  |  |  |  |  |
| **5+ previous inpatient episodes** | 0.65  (0.30-1.41)  p = 0.275 | 1.00  (0.98-1.02)  p = 0.964 | 0.79  (0.40-1.58)  p = 0.509 | 1.21  (0.76-1.94)  p = 0.429 | 0.80 (0.36-1.78); 0.587 | 1.17  (0.76-1.81)  p=0.474 | 0.97  (0.54-1.75)  p = 0.924 | 1.08  (0.70-1.65)  p = 0.736 | 2.00  (0.78-5.13)  p = 0.147 |  |  |
|  |  |  |  |  | 0.82 (0.19-3.60); 0.789 |  |  |  |  |  |  |
|  |  |  |  |  | 2.55 (1.05-6.18); 0.039 |  |  |  |  |  |  |
|  |  |  |  |  | 1.22 (0.77-1.93); 0.396 |  |  |  |  |  |  |
| **Lifetime DUD** | 0.50  (0.23-1.07)  p=0.075 | 1.00  (0.98-1.02)  p = 0.714 | 1.19  (0.61-2.31)  p = 0.615 | 1.09  (0.70-1.71)  p = 0.690 | 1.67 (0.78-3.61); 0.189 | n/a | 1.19  (0.66-2.16)  p = 0.567 | 1.09  (0.73-1.64)  p = 0.672 | 0.90  (0.37-2.17)  p = 0.809 | 0.81  (0.53-1.25)  p = 0.346 |  |
|  |  |  |  |  | 0.82 (0.33-2.05); 0.673 |  |  |  |  |  |  |
|  |  |  |  |  | 3.54 (1.46-8.56); 0.005 |  |  |  |  |  |  |
|  |  |  |  |  | 1.42 (0.91-2.22); 0.122 |  |  |  |  |  |  |
| **Length of stay** | 0.79  (0.34-1.83)  p=0.579 | 0.99  (0.97-1.01)  p = 0.496 | 0.89  (0.41-1.93)  p = 0.772 | 1.41  (0.92-2.17)  p = 0.115 | 1.65 (1.03-2.63); 0.037 | 0.90  (0.59-1.36)  p = 0.603 | 0.79  (0.46-1.38)  p = 0.408 | 1.02  (0.67-1.54)  p = 0.934 | 0.72  (0.26-1.99)  p = 0.532 | 1.01  (0.66-1.53)  p = 0.981 | 0.96  (0.64-1.44)  0.845 |
|  |  |  |  |  | 1.06 (0.59-1.89); 0.852 |  |  |  |  |  |  |
|  |  |  |  |  | 1.02 (0.62-1.67); 0.950 |  |  |  |  |  |  |
|  |  |  |  |  | 1.33 (1.00-1.78); 0.052 |  |  |  |  |  |  |

Note: Bonferoni-adjusted significance level of p=0.0005 (p = 0.05 / 97 analyses)

**Appendix table 6 Model coefficients**

| **Variable** | **Model coefficient** |
| --- | --- |
| Sex (female) | -0.0299 |
| Age at discharge | -0.8407 |
| Previous violent crime | 1.1682 |
| Previous serious violent crime | -0.4480 |
| Primary diagnosis at discharge   - Schizophrenia spectrum - Bipolar disorder - Unipolar depression - Anxiety disorders - Other | 0  0.5994  0.2867  0.1142  0.304 |
| Drug use disorder at hospitalisation or discharge | -0.1188 |
| Alcohol use disorder at hospitalisation or discharge | 0.2288 |
| Personality disorder at discharge | 0.3052 |
| Employment before admission | -0.578 |
| Number of previous inpatient episodes (five or more) | -0.4676 |
| Lifetime drug use disorder | 0.7964 |
| Length of stay in forensic hospital (12 months or more) | -0.4576 |

LC (linear conbimation)=∑ beta*value of risk factor

Risk of violent offending within 12 months = 1 – 0.9280^exp(LC)

Risk of violent offending within 24 months = 1 – 0.8762^exp(LC)

Tripod Checklist

| **Section/Topic** | **Item** |  | **Checklist Item** | **Page** |
| --- | --- | --- | --- | --- |
| **Title and abstract** | | | | |
| Title | 1 | D;V | Identify the study as developing and/or validating a multivariable prediction model, the target population, and the outcome to be predicted. | 1 |
| Abstract | 2 | D;V | Provide a summary of objectives, study design, setting, participants, sample size, predictors, outcome, statistical analysis, results, and conclusions. | 2 |
| **Introduction** | | | | |
| Background and objectives | 3a | D;V | Explain the medical context (including whether diagnostic or prognostic) and rationale for developing or validating the multivariable prediction model, including references to existing models. | 3 |
|  | 3b | D;V | Specify the objectives, including whether the study describes the development or validation of the model or both. | 3 |
| **Methods** | | | | |
| Source of data | 4a | D;V | Describe the study design or source of data (e.g., randomized trial, cohort, or registry data), separately for the development and validation data sets, if applicable. | 4 |
|  | 4b | D;V | Specify the key study dates, including start of accrual; end of accrual; and, if applicable, end of follow-up. | 4 |
| Participants | 5a | D;V | Specify key elements of the study setting (e.g., primary care, secondary care, general population) including number and location of centres. | 4 |
|  | 5b | D;V | Describe eligibility criteria for participants. | 4 |
|  | 5c | D;V | Give details of treatments received, if relevant. | 4 |
| Outcome | 6a | D;V | Clearly define the outcome that is predicted by the prediction model, including how and when assessed. | 4 |
|  | 6b | D;V | Report any actions to blind assessment of the outcome to be predicted. | 5 |
| Predictors | 7a | D;V | Clearly define all predictors used in developing or validating the multivariable prediction model, including how and when they were measured. | 4, A |
|  | 7b | D;V | Report any actions to blind assessment of predictors for the outcome and other predictors. | 4, A |
| Sample size | 8 | D;V | Explain how the study size was arrived at. | 4 |
| Missing data | 9 | D;V | Describe how missing data were handled (e.g., complete-case analysis, single imputation, multiple imputation) with details of any imputation method. | 5 |
| Statistical analysis methods | 10a | D | Describe how predictors were handled in the analyses. | 5, A |
|  | 10b | D | Specify type of model, all model-building procedures (including any predictor selection), and method for internal validation. | 4-6, A |
|  | 10c | V | For validation, describe how the predictions were calculated. | 6, A |
|  | 10d | D;V | Specify all measures used to assess model performance and, if relevant, to compare multiple models. | 6, A |
|  | 10e | V | Describe any model updating (e.g., recalibration) arising from the validation, if done. | - |
| Risk groups | 11 | D;V | Provide details on how risk groups were created, if done. | 6, A |
| Development vs. validation | 12 | V | For validation, identify any differences from the development data in setting, eligibility criteria, outcome, and predictors. | - |
| **Results** | | | | |
| Participants | 13a | D;V | Describe the flow of participants through the study, including the number of participants with and without the outcome and, if applicable, a summary of the follow-up time. A diagram may be helpful. | 7, T |
|  | 13b | D;V | Describe the characteristics of the participants (basic demographics, clinical features, available predictors), including the number of participants with missing data for predictors and outcome. | 7, T |
|  | 13c | V | For validation, show a comparison with the development data of the distribution of important variables (demographics, predictors and outcome). | - |
| Model development | 14a | D | Specify the number of participants and outcome events in each analysis. | 7, T |
|  | 14b | D | If done, report the unadjusted association between each candidate predictor and outcome. | - |
| Model specification | 15a | D | Present the full prediction model to allow predictions for individuals (i.e., all regression coefficients, and model intercept or baseline survival at a given time point). | T, A |
|  | 15b | D | Explain how to the use the prediction model. | 8, A |
| Model performance | 16 | D;V | Report performance measures (with CIs) for the prediction model. | 7-8, AT |
| Model-updating | 17 | V | If done, report the results from any model updating (i.e., model specification, model performance). | - |
| **Discussion** | | | | |
| Limitations | 18 | D;V | Discuss any limitations of the study (such as nonrepresentative sample, few events per predictor, missing data). | 10 |
| Interpretation | 19a | V | For validation, discuss the results with reference to performance in the development data, and any other validation data. | - |
|  | 19b | D;V | Give an overall interpretation of the results, considering objectives, limitations, results from similar studies, and other relevant evidence. | 9-11 |
| Implications | 20 | D;V | Discuss the potential clinical use of the model and implications for future research. | 9-11 |
| **Other information** | | | | |
| Supplementary information | 21 | D;V | Provide information about the availability of supplementary resources, such as study protocol, Web calculator, and data sets. | A |
| Funding | 22 | D;V | Give the source of funding and the role of the funders for the present study. | 2 |

*Items relevant only to the development of a prediction model are denoted by D, items relating solely to a validation of a prediction model are denoted by V, and items relating to both are denoted D;V. We recommend using the TRIPOD Checklist in conjunction with the TRIPOD Explanation and Elaboration document.
A: Appendix; T: Tables
